# Bitumen Binders Modified with Sulfur/Organic Copolymers

**DOI:** 10.3390/ma15051774

**Published:** 2022-02-26

**Authors:** Jakub Wręczycki, Yuriy Demchuk, Dariusz M. Bieliński, Michael Bratychak, Volodymyr Gunka, Rafał Anyszka, Tomasz Gozdek

**Affiliations:** 1 Institute of Polymer and Dye Technology, Faculty of Chemistry, Lodz University of Technology, 16 Stefanowskiego Street, 90-537 Lodz, Poland; dariusz.bielinski@p.lodz.pl (D.M.B.); rafal.anyszka@p.lodz.pl (R.A.); tomasz.gozdek@p.lodz.pl (T.G.); 2 Department of Chemical Technology of Oil and Gas Processing, Institute of Chemistry and Chemical Technologies, Lviv Polytechnic National University, 12 Bandery Street, 79013 Lviv, Ukraine; mbratychak@gmail.com (M.B.); vgunka@gmail.com (V.G.)

**Keywords:** polysulfides, sulfur polymers, inverse vulcanization, polymer-modified bitumen

## Abstract

With the continuing growth of waste sulfur production from the petroleum industry processes, its utilization for the production of useful, low-cost, and environmentally beneficial materials is of primary interest. Elemental sulfur has a significant and established history in the modification of bitumen binders, while the sulfur-containing high-molecular compounds are limited in this field. Herein, we report a novel possibility to utilize the sulfur/organic copolymers obtained via the inverse vulcanization process as modifiers for bitumen binders. Synthesis and thermal characterization (TGA-DSC) of polysulfides derived from elemental sulfur (S_8_) and unsaturated organic species (dicyclopentadiene, styrene, and limonene) have been carried out. The performance of modified bitumen binders has been studied by several mechanical measurements (softening point, ductility, penetration at 25 °C, frass breaking point, adhesion to glass and gravel) and compared to the unmodified bitumen from the perspective of normalized requirements concerning polymer-modified bitumen. The interaction of bitumen binder with sulfur/organic modifier has been studied by means of FTIR spectroscopy and DSC measurements. The impact of the modification on the performance properties of bitumen has been demonstrated. The bitumen binders modified with sulfur/organic copolymers are in general less sensitive to higher temperatures (higher softening point up to 7 °C), more resistant to permanent deformations (lower penetration depth), and more resistant to aging processes without intrusive deterioration of parameters at lower temperatures. What is more, the modification resulted in significantly higher adhesion of bitumen binders to both glass (from 25% up to 87%) and gravel surfaces in combination with a lower tendency to form permanent deformations (more elastic behavior of the modified materials).

## 1. Introduction

The utilization of sulfur excess, mainly manufactured as a side product of the petroleum industry processes, constitutes an important and challenging problem for the chemical industry and related branches [1,2]. Exploration of low-cost, high-sulfur content organic or polymeric materials fabricated from elemental sulfur (S_8_) and its further utilization pathways should be therefore of primary interest to sustain the proper cycle of sulfur conversion [2,3]. Recently, remarkable progress and renewed interest have been observed in elemental sulfur-based polymer chemistry. The scope of possibilities, accurate comparisons of the polysulfides synthesis methods as well as the green aspects of inclusion of elemental sulfur to polymer chemistry was summarized in exemplary comprehensive reviews [4,5,6].

The addition of high-molecular materials to a raw bitumen binder constitutes a very promising trend in the modification of its properties nowadays. The modification makes fatigue resistance of bitumen extended, its stiffness at higher temperatures increased, or cracking resistance at lower temperatures higher [7,8,9]. The rapidly growing pavement industry in the last few decades and, consequently, increased vehicular traffic load, forced the attempts of quality improvement of the most sensitive component of asphalt concrete [7,10]. Therefore, various polymers have been utilized over the years as bitumen binder modifiers with an example of thermoplastic polymers (polyethylene [11], polypropylene [12], ethylene vinyl acetate or ethylene butyl acrylate [13]), thermoplastic elastomers (styrene-butadiene-styrene triblock copolymers (SBS) [13,14]), polycondensation resins (phenol-cresol-formaldehyde resins and phenol-formaldehyde resins with labile peroxy bonds or methacrylic components [10,15]), petroleum resins with epoxy, hydroxy or carboxy groups [16], low-molecular organic compounds (formaldehyde and maleic anhydride) [17,18], or recycled elastomers in the form of ground tire rubber [19].

Historically speaking, a combination of bitumen binders with elemental sulfur as modifier started in the 19th century, but the first practical tests had been carried out and described later on, at the end of the 1930s by Bencowitz and Boe [20]. Although, the results were relatively successful, the costs of sulfur-overpriced bitumen binder resulted in discontinuing the research for the next decades. The real interest in the utilization of sulfur in asphalt paving materials was recognized in the 1970s and 1980s as a result of the growing generation of recovered sulfur from secondary sources and, consequently, a sulfur price decrease [21,22,23]. Primarily, the addition of large amounts of sulfur to asphalt concrete was intended to partially replace bitumen binder and decrease the costs of asphalt concrete. The formed materials were so-called “sulfur-extended asphalts” (SEA) and offered generally comparable or even superior properties compared to conventional asphalt concrete [23,24]. The upgraded technology of SEA is constantly in use and development under the name of Shell Thiopave^®^ (Shell Brands International AG, Baar, Switzerland), offering a pelleted form of plasticized sulfur, which significantly reduces the hydrogen sulfide and sulfur dioxide emissions during mixing, and enables asphalt production at lower temperatures [24].

Sulfur as a modifier in smaller quantities also was investigated in the absence [25,26,27] or the presence of other additives, predominantly of rubbers such as natural or synthetic rubbers [8,28] or SBS [8,29]. In the latter case, the presence of sulfur in the rubber-modified bitumen may enable interconnections through sulfide or polysulfide links between polymer and bitumen via vulcanization of both unsaturated rubber and functionalized bitumen components improving its storage stability and rheological properties such as resistance to deformation [7,8].

Surprisingly, not enough attention has been focused on using a polymerized form of sulfur and other high-molecular sulfur-containing compounds in bitumen binder modification. Besides the work of Elkholy et al. [30], who used the polysulfides derived from elemental sulfur thermally modified with a mixture of residual olefinic hydrocarbons (distillate fractions of C_5_) and bituminous residue as a modifier of bitumen binders and the work of Liang et al. [31], concerning the effect of polymerized sulfur on SBS-modified asphalt, no other reports are present in the literature. This conceives a new potential area to utilize sulfur/organic copolymers.

In order to fulfill this research gap as well as encourage by successful application of sulfur/organic copolymers obtained via the “inverse vulcanization” process as curing agents for rubber in our previous work [32], herein we report the application of selected polysulfides of this type as modifiers for bitumen binder. The thermal properties of sulfur/organic copolymers as well as their content optimization from the point of their performance as bitumen modifiers were evaluated. The characteristics of unmodified and modified bitumen samples were compared in terms of mechanical parameters and adhesive properties of bitumen binders.

## 2. Materials and Methods

### 2.1. Materials

Elemental sulfur (S_8_), in a form of powder, was received from Siarkopol Tarnobrzeg (Tarnobrzeg, Poland). Dicyclopentadiene (DCPD) (≥95%) was purchased from Merck KGaA (Darmstadt, Germany), styrene (ST) (99%) from Acros Organics (Geel, Belgium), while the limonene (LIM) was synthesized (extracted from orange peel skin) from the Institute of Organic Chemistry (Lodz University of Technology, Lodz, Poland). The bitumen binder amenable to modification BND 60/90 was produced by PJSC “Transnational financial and industrial oil company Ukrtatnafta” (Kremenchuk, Ukraine).

### 2.2. Structure of the Experimental Work

**Scheme 1:**
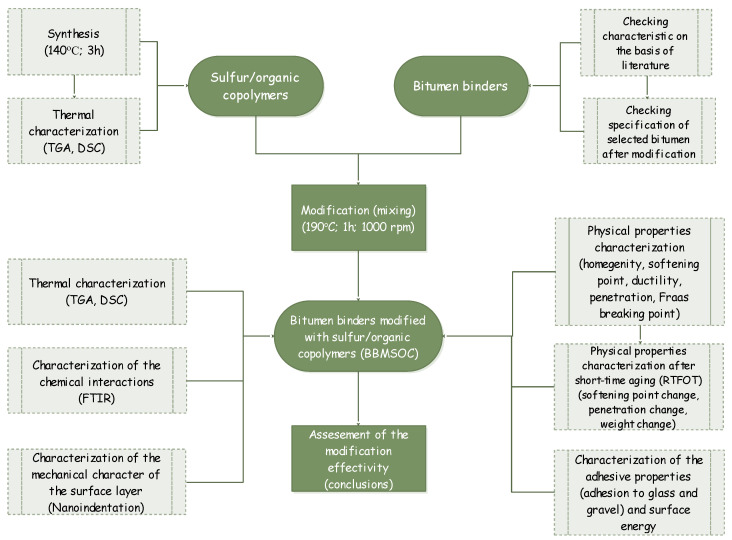
A schematic flow chart of the carried-out experimental work.

The research framework including particular experimental steps has been presented in Scheme 1. The comprehensive descriptions of selected steps and methods are presented below in Section 2.3, Section 2.4, Section 2.5, Section 2.6, Section 2.7, Section 2.8, Section 2.9 and Section 2.10.

### 2.3. Synthesis of Sulfur/Organic Copolymers

The synthesis of the sulfur/organic copolymers was carried out in a glass reaction flask (250 mL volume), covered with a four-necked glass lid. The glass lid was equipped with a condenser, dropping funnel, mechanical stirrer, and a thermocouple. The reaction vessel with sulfur inside was immersed in a silicone oil bath and heated up to the temperature of 140 °C. While heating, at a temperature point around 120 °C, the sulfur started to melt down, and the mechanical stirrer was launched. After the temperature hit the final temperature setpoint, the molten sulfur was stirred for approximately 30 min. Afterward, the organic comonomers were dripped into the liquid sulfur within 30 min each time. Due to the exothermic reaction, the temperature was supervised to not exceed 160 °C. The copolymerization process in a bulk (known in this case as an “inverse vulcanization”) was carried out under the air atmosphere and atmospheric pressure. The obtained products were poured out into silicone molds at room temperature and solidified. Before its application, the copolymers were crushed with the use of mortar and pestle to obtain the powdery materials used further as bitumen binder modifiers. Copolymerization conditions and composition of the sulfur/organic copolymers are summarized in Table 1.

### 2.4. Thermal Analysis of Sulfur/Organic Copolymers

The thermogravimetric analysis (TGA) and differential scanning calorimetry (DSC) of the sulfur/organic copolymers were carried out using Netzsch TG 209 and Netzsch DSC 204 instruments (Netzsch, Selb, Germany), respectively. Both analyzers were connected with a Netzsch TASC 414/3A heat controller (Netzsch, Selb, Germany). The TGA tests were performed under nitrogen (N_2_) atmosphere in aluminum crucibles on a small powder sample (9–11 mg). The tested temperature range was 40–450 °C, applying heating mode and a rate of 10 °C/min. The DSC analyses were performed only in a heating mode under nitrogen (N_2_) atmosphere in the temperature range from 40 to 200 °C. The samples were tested in aluminum crucibles with variable heating rates. In the temperature range from 40 to 80 °C and from 140 to 200 °C, the heating rate was set to 7.5 °C/min, while in the temperature range from 80 to 140 °C, the heating rate was set to 2.5 °C/min. The lower heating rate in this temperature range was applied to provide better peaks resolution that occurred in this range.

### 2.5. Modification of the Bitumen Binders

Modifications of the bitumen were carried out in a glass modification vessel equipped with a mechanical stirrer and a thermometer, placed on a laboratory hotplate. The definite amount of bitumen BND 60/90 was heated up to the appropriate temperature (190 °C), and afterward, the particular modifier (sulfur/organic copolymer) was added. The amount of sulfur/organic copolymers as a modifier (1.0; 2.5 wt %) was pre-investigated experimentally. The mixture was stirred for 1 h at 1000 rpm.

### 2.6. Analysis of Physical Properties of Bitumen Binders

Analysis of the unmodified (raw) bitumen binder and the bitumen binders modified with sulfur/organic copolymers (BBMSOC) were investigated according to the European standard test methods dedicated to the bitumen and the asphalt binders (PN-EN) as well as to the Ukrainian national standard test procedures (DSTU). The BBMSOC were compared either with unmodified (raw) bitumen BND 60/90 as well as with the requirements for the performance of oil-oxidized polymer-modified bitumen binder (used as a reference) with softening point equal at least to 53 °C for 60/90-53 bitumen, regulated by the Ukrainian national standard DSTU B V.2.7-135:2014 [33]. The softening point of unmodified and modified bitumen binders was determined with the use of the ring and ball method, according to the PN-EN 1427:2015 European standard test method [34]. The needle penetration at 25 °C and the penetration index were investigated according to the PN-EN 1426:2015 [35] and PN-EN 12591:2009 [36] European standard test methods, respectively. Ductility at 25 °C was measured according to the PN-EN 13587:2010 [37] standard and the Fraas breaking point of prepared bitumen binders, as stated in the PN-EN 12593:2015 standard [38]. Adhesion to the surface of the glass was assessed according to the Ukrainian standard test method DSTU B V.2.7-81-98 [39] with a supplement according to the point 8.6 of the DSTU 4044-2001 standard [40], while adhesion to the gravel surface according to point 28 of the DSTU B V.2.7-89-99 standard [41]. Homogeneity was determined as follows: the sample of 300 ÷ 350 g of BBMSOC was heated up to the temperature of 160 ± 5 °C and stirred for 10 ÷ 15 min. After that, the bitumen binder was decanted to the porcelain vessel and a glass rod was immersed in the bitumen at the depth of 6 ÷ 8 cm and pulled out afterward. The organoleptic assessment of bitumen downflow was done; if the surface of the rod is homogenously coated without particles of unsolved modifier, the homogeneity of bitumen would be accepted (+). The following procedure was repeated at least 3 times for each bitumen sample. Short-term aging was simulated using the RTFOT (Rolling Thin Film Oven Test). In this study, the test was carried out in accordance with the PN-EN 12607-1 European standard test method for 75 min, at 163 °C [42].

### 2.7. FTIR Measurements of Bitumen Binders

The Fourier transform infrared (FTIR) spectroscopy studies were performed at room temperature by using a Thermo Scientific Nicolet 6700 FTIR spectrometer equipped with a diamond Smart Orbit ATR sampling accessory (Thermo Fisher Scientific, Waltham, MA, USA). The FTIR absorbance spectra were investigated in the absorption mode (64 scans) within the wavenumber range of 4000–400 cm^−1^ with the spectral resolution set to 4 cm^−1^.

### 2.8. Thermal Analysis of Bitumen Binders

The differential scanning calorimetry (DSC) and thermogravimetric analysis (TGA) of bitumen binders were carried out by using a Mettler Toledo TGA/DSC 1 STARe System (Mettler Toledo, Greifensee, Switzerland) equipped with a Gas Controller GC10 (Mettler Toledo, Greifensee, Switzerland). The DSC samples were firstly cooled from room temperature to −90 °C with a cooling rate of 10 °C/min and afterward heated up and investigated in a temperature range from −80 °C to 200 °C, with a heating rate of 10 °C/min. The TGA tests were carried out with a heating rate of 10 °C/min in a temperature range from 25 °C to 600 °C under an argon atmosphere.

### 2.9. Nanoindentation Test of Bitumen Binders

The microhardness characteristic of the composites was tested with a NanoTest 600 instrument (Micromaterials Ltd., Wrexham, UK) equipped with a Berkovich indenter, using depth controlled (3000 nm) mode with the loading/unloading speed of dP/dt = 0.05 mN/s. The measurements were carried out under controlled conditions: temperature 20 ± 2 °C and relative humidity of 60 ± 5%. The characteristic of the composite surface was calculated based on the unloading curve, using the procedure proposed by Olivier and Pharr [43].

### 2.10. Surface Free Energy of Bitumen Binders

The sessile drop method was applied for contact angle measurements in order to calculate the surface free energy (SFE) of bitumen binders. The samples were poured onto glass plates. Two liquids differing in polarity: distilled water and 1,4-dioodomethane were applied in a form of 2 µL droplets under ambient conditions. An OCA 15EC goniometer (DataPhysics Instruments GmbH, Filderstadt, Germany) equipped with a single direct dosing system (0.01–1 mL B. Braun syringe, Melsungen, Germany) was used. To calculate SFE of the bitumen binders, the Owens–Wendt–Rabel–Kaelble method was utilized [44] according to the formulas presented below:

The surface free energy is the sum of two components: (1) dispersive (*γ^d^_s_*) and polar components (*γ^p^_s_*), Equation (1).

(1) $$\gamma_{s}^{}=\gamma_{s}^{d}+\gamma_{s}^{p}$$

The dispersive component can be determined from the formula below, Equation (2):

(2) $$\gamma_{s}^{d}={\left[\frac{\sqrt{\gamma_{1}^{p}}\cdot \gamma_{2}\cdot (\cos\theta_{2}+1)-\sqrt{\gamma_{2}^{p}}\cdot \gamma_{1}\cdot (\cos\theta_{1}+1)}{2\cdot (\sqrt{\gamma_{2}^{d}\cdot \gamma_{1}^{p}}-\sqrt{\gamma_{2}^{p}\cdot \gamma_{1}^{d}})}\right]}^{2}$$

While the value of the polar component can be determined from the formula below, Equation (3):

(3) $$\gamma_{s}^{p}={\left[\frac{\gamma_{1}\cdot (\cos\theta_{1}+1)-2\cdot \sqrt{\gamma_{1}^{d}\cdot \gamma_{s}^{d}}}{\sqrt{\gamma_{1}^{d}}}\right]}^{2}$$

where: *γ^p^*_1,_ *γ^d^*_1,_ *γ*_1_—consecutively: polar and dispersion components and their sum for a polar liquid; *γ^p^*_2,_ *γ^d^*_2,_ *γ*_2_—consecutively: polar and dispersion components and their sum for a non-polar liquid; *θ*_1_, *θ*_2_ contact angle of a polar and non-polar liquid, respectively.

The surface tensions and their components for low molecular weight liquids (mJ/m^2^), which were used for the Owens–Wendt calculations: water (polar liquid) (*γ^p^* = 51.0, *γ^d^* = 21.6, *γ* = 72.6), diiodomethane (non-polar liquid) (*γ^p^* = 2.3, *γ^d^* = 48.5, *γ* = 50.8)

## 3. Results and Discussion

### 3.1. Thermal Behaviour of Sulfur/Organic Copolymers (TGA-DSC)

**Figure 1:**
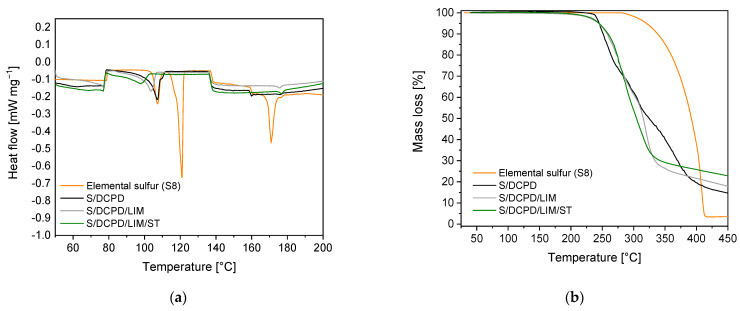
Thermal analysis curves of sulfur/organic copolymers presented by: (**a**) differential scanning calorimetry (DSC); (**b**) thermogravimetry (TGA). The curves of elemental sulfur were added for comparison.

The investigation of sulfur/organic copolymers using differential scanning calorimetry (DSC) and thermogravimetry (TGA) was aimed to analyze their behavior at the temperatures required in the bitumen modification process (190 °C). The calorimetry provided information concerning their phase structure, while the thermogravimetric studies determined the thermal stability of the copolymers. Elemental sulfur (S_8_) curves were added for comparison. The onset decomposition temperature was established as a temperature required for 5% mass loss during decomposition (T_5%_). The results are presented in Figure 1a,b and summarized in Table 2.

The DSC curve of elemental sulfur is represented by three distinct peaks with a maximum at 108 °C, 120 °C, and 170 °C, corresponding, respectively, to (i) the transition of crystalline structure, namely from orthorhombic sulfur to monoclinic sulfur, (ii) melting of the sulfur crystals and (iii) the latter to the ring-opening polymerization of sulfur molecules. Non-purified sulfur/organic copolymers are composed of macromolecules (polymeric phase) and elemental sulfur unreacted in the (co)polymerization process (crystalline phase). In this case, the DSC curve reveals one broader peak in the temperature interval from c.a. 90 °C to 110 °C. It may be ascribed to the melting of the residual low-molecular crystalline sulfur disrupted by sulfur/organic macromolecules existing in solid solution mixture (fused melting) [45,46]. The enthalpy values of this effect significantly diversify depending on the copolymer type. Also, the peaks are shifted into lower temperatures in the case of S/DCPD/LIM and S/DCPD/LIM/ST copolymers. According to our previous investigation [32], purification of copolymers by solvent extraction, from elemental sulfur, should lead to these peaks disappearing. Generally, the lower enthalpy of the melting peak, the lower elemental sulfur (crystalline) content in sulfur/organic copolymers, and simultaneously the higher polymeric phase (amorphous) content [45]. Following this rule, the highest polymeric phase content, and the lowest content of S_8_ possess S/DCPD/LIM/ST copolymer (ΔH = 14.7 J/g). In the case of sulfur/organic copolymers, peaks characteristics for the ring-opening polymerization of the residual sulfur are very small and their enthalpy is negligible. This may tentatively confirm the possibility of unreacted sulfur molecules trapping in the solid solution.

The TGA curves of sulfur/organic copolymers reveal non-uniform and non-stable kinetics during thermal decomposition, compared to pure elemental sulfur (S_8_). The onset temperature is also significantly lower, which is a general affliction of polysulfide polymers [47] and starts at c.a. 245 °C, whereas elemental sulfur at 320 °C. The kinetics of thermal decomposition is influenced significantly by the presence of unreacted sulfur and the presence of organic fractions originating from used comonomers. What is more, the distribution of polymeric phase and crystalline sulfur in the structure of the copolymers are unknown on the micromorphological level and the structure of sulfur/organic copolymers is not well characterized in this term. According to the TGA results, sulfur/organic copolymers may be utilized in the selected temperature applications such as bitumen modification (190 °C) or rubber crosslinking (160 °C) [32] without intrusive degradation.

### 3.2. Properties of Bitumen Binders Modified with Sulfur/Organic Copolymers (BBMSOC)

#### 3.2.1. Physical Properties of BBMSOC

**Figure 2:**
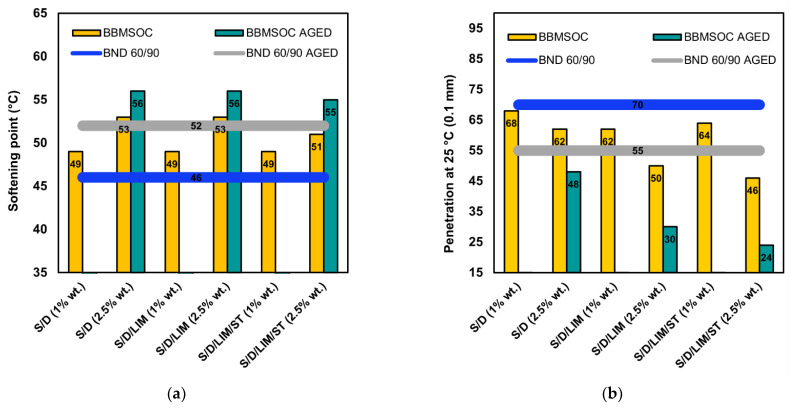

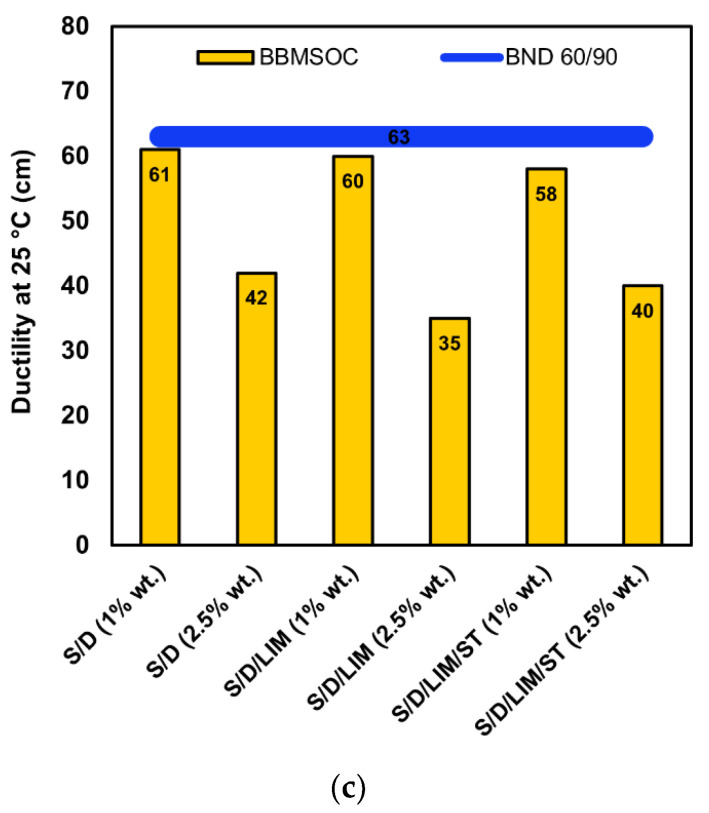
Influence of sulfur/organic copolymers addition on the performance parameters of bitumen binders (BBMSOC) before and after RTFOT aging: (**a**) softening point; (**b**) penetration; (**c**) ductility.

Performance of the modified bitumen binders containing variable content of different sulfur/organic copolymers as modifiers were investigated and compared to the values of unmodified bitumen binder sample (BND 60/90) and standardized oil-oxidized polymer-modified bitumen (BMPA 60/90-53). The results obtained are shown in Table 3 and are presented in Figure 2a–c. The results of short-term RTFOT aging of the unmodified bitumen and modified bitumen binders containing 2.5% wt sulfur/organic copolymers are presented in the form of a comparative Table 4.

The incorporation of sulfur/organic copolymers either in 1.0% wt or 2.5% wt into BND 60/90 bitumen resulted in homogeneous polymer–bitumen blends. As can be noticed based on Table 3 and Figure 2a–c, the performance parameters of the prepared BBMSOC differ significantly in comparison to the raw BND 60/90 bitumen binder. Regardless of sulfur/copolymer type, their addition increases the softening temperature and decreases penetration without significant impact on the behavior of binders in low temperatures (Fraas breaking point). A similar general trend of softening point and penetration changes has been obtained by Elkholy et al. [30], who used the thermally modified sulfur (90% wt) with a mixture of residual olefinic hydrocarbons (distillate fractions of C_5_) and bituminous residue (10% wt) as a modifier of bitumen binder (60/70). In turn, modification of BND 60/90 with elemental sulfur carried out by Syroezhko et al. [25] with small sulfur addition (below 10% wt) led to a decrease of softening point and increase of penetration (plasticization effect), while above 10% wt (up to 40 wt %) surprisingly reversed the trend.

The highest increase of softening point (+7 °C) has been observed in the case of S/DCPD and S/DCPD/LIM copolymers within 2.5% wt addition, which fulfills the softening point of standardized BMPA 60/90-53 bitumen. On the other hand, a decrease in penetration is significant, particularly with the addition of higher amounts of sulfur/organic copolymers. In the case of 2.5% wt addition of S/DCPD/LIM and S/DCPD/LIM/ST, penetration values were strongly reduced and went below the standardized values (61–90) of BMPA 60/90-53. Only the sample S/DCPD (2.5% wt) shows an acceptable value. The rigid character of samples with additive above 1.0% wt of the corresponding modifier can be also confirmed by the values of ductility at 25 °C, which were notably reduced compared to 1.0% wt additive as well as to unmodified bitumen, even up to 28 cm in the case of bitumen binder modified with S/DCPD/LIM copolymer. Overall, the modification enables to retain of the properties of bitumen binders in low temperatures, which is an additive value. The simulation of the RTFOT aging demonstrates an acceptable process, according to the specification contained in the PN-EN 12591 European standard test [36]. During aging, the bitumen penetration decreases and its softening temperature increases. The best example for short-term aging using RTFOT exhibits bitumen BND 60/90 with 2.5% wt addition of S/DCPD, because it has a high value of residual penetration (77%) together with the lowest change to weight (0.03 wt %) and to softening point (3 °C). The increase of softening point after aging in the case of raw BND 60/90 is 6 °C while in the case of the modified systems is 3 °C (S/DCPD and S/DCPD/LIM) or 4 °C (S/DCPD/LIM/ST). The lower increase indicates a higher resistance to aging processes.

For selected modification systems such as S/DCPD (2.5% wt), that fall within all the required parameters of standardized BMPA 60/90-53 bitumen, the presented results may be attractive to produce paving materials of less sensitivity to higher temperatures and loading time exposure, also presenting increased resistance to permanent deformations without intrusive deterioration of parameters at lower temperatures. Selected tested modification systems significantly increase the performance of bitumen binder compared to raw BND 60/90 and fulfill the majority of the comparative requirements (including adhesion presented in point 3.3.3). Some modification systems, unfortunately, do not fall within one parameter for the BMPA 60/90-53, such as softening point (S/DCPD (1.0% wt), S/DCPD/LIM (1.0% wt), and S/DCPD/LIM/ST (1.0% wt)), or penetration (S/DCPD/LIM (2.5% wt)). These limitations should be taken into consideration.

#### 3.2.2. Nanoindentation Results

**Figure 3:**
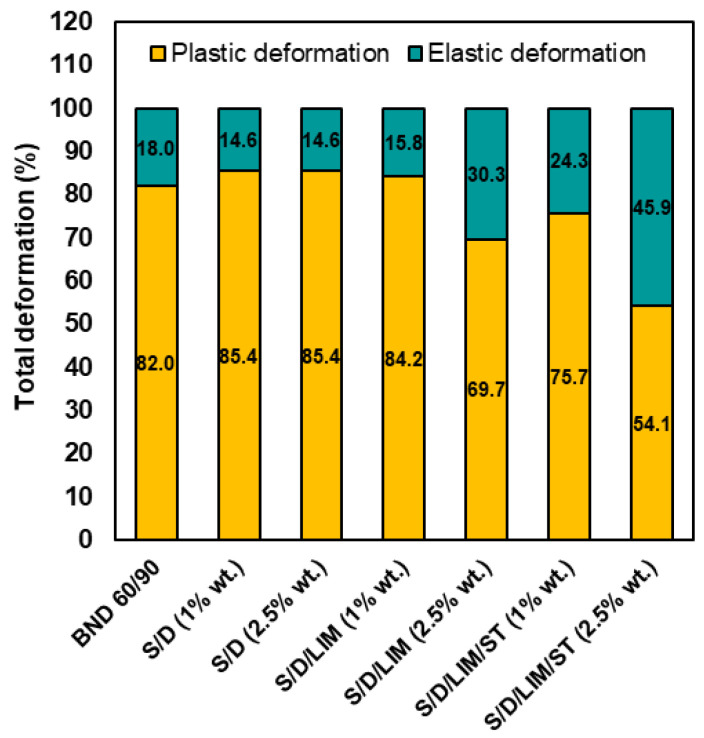
Participation of plastic and elastic deformation in the total samples deformation of BBMSOC.

The nanoindentation investigation of BBMSOC has been performed to determine the plastic part of the sample deformation. This is to compare the potential behavior of the bitumen binders studied to rutting. The results obtained are shown in Figure 3.

Nanoindentation measurements make it possible to determine the mechanical character of the surface layer of the materials. The incorporation of S/DCPD copolymer neither in 1 wt % nor in 2.5 wt % does not significantly change the mechanical characteristic of the bitumen surface compared to the reference BND 60/90. A minor increase in plastic deformation of the surface layer can be the result of the plasticization effect. It may be ascribed to the structure of the copolymer, which contains only dicyclopentadiene units along the macromolecules and presumably is unable to form crosslinked structures. The more structurally rich materials, such as S/DCPD/LIM and S/DCPD/LIM/ST copolymers, which contain units of limonene or limonene and styrene, respectively, may form more extended polymer networks during the inverse vulcanization process and added to the bitumen composition interpenetrate the network formed by high-molecular bitumen components such as asphaltenes. This may result in a more elastic behavior of the surface layer. It can be noticed that this effect increases with an increase in the addition of the copolymer in the case of S/DCPD/LIM and S/DCPD/LIM/ST modifiers. The highest effect is observed for the S/DCPD/LIM/ST within the addition of 2.5 wt %, making the elastic surface deformation of BBMSOC increase over 2.5 times compared to the unmodified BND 60/90 bitumen. Observed relationships are consistent with the penetration results trend. The bitumen binders after modification with the use of S/DCPD/LIM and S/DCPD/LIM/ST copolymers are more rigid and simultaneously of more elastic character. The nanoindentation results indicate that the application of selected sulfur/organic copolymers to raw bitumen binder may be attractive as potential successful rutting preventing modifiers.

#### 3.2.3. Adhesive Properties and Surface Energy of BBMSOC

Adhesion of bitumen binders to the so-called “acidic” aggregates (aggregates with high content of silica) such as quartzite or granite is imperfect [10]. On the basis of that, many scientific groups and industrial companies are developing new bitumen promoters to enhance the adhesion of bitumen to aggregates and consequently increase the water-resistance of pavements [48].

**Figure 4:**
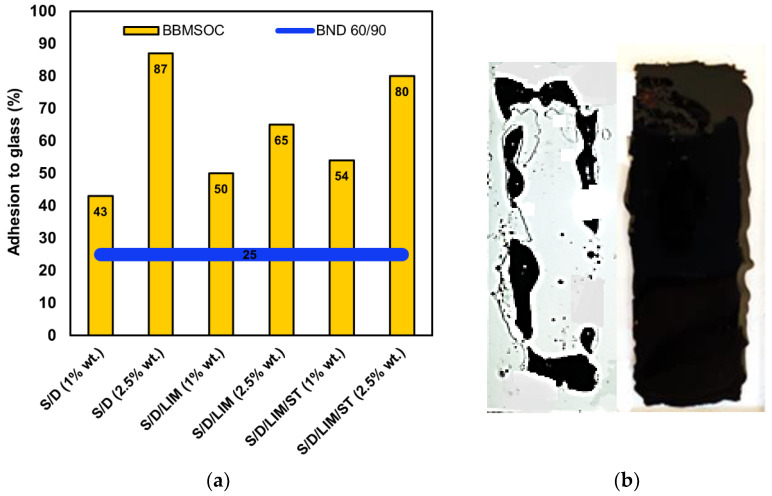

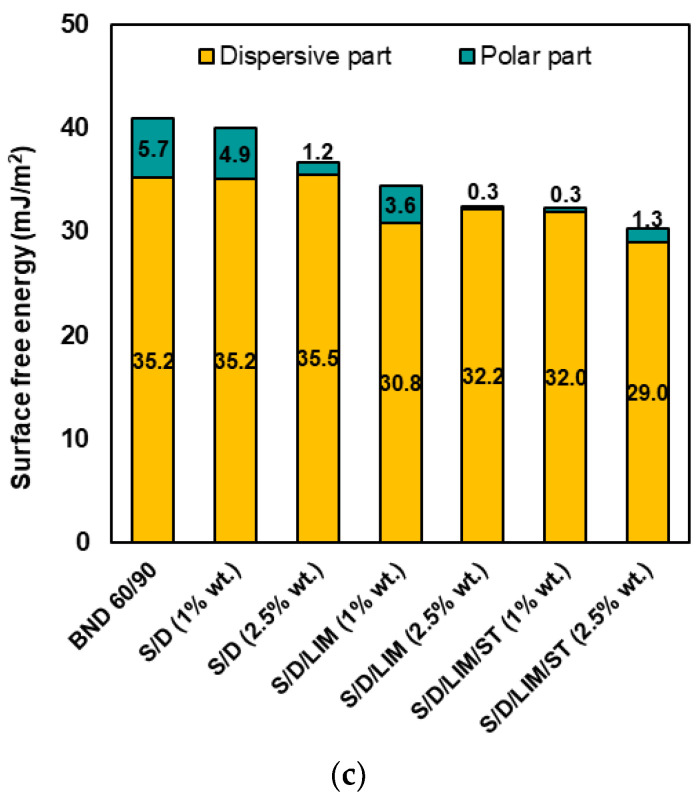
The results of (**a**) adhesion to the glass surface in graph form; (**b**) pictures of BND 60/90 (adhesion 25%) (**left**); BND 60/90 with and 2.5% wt addition of S/DCPD copolymer (adhesion 87%) (**right**); (**c**) participation of dispersive and polar part in the total surface free energy of BBMSOC.

In our study, an investigation of the adhesive properties of sulfur/organic copolymers-modified bitumen binders to granite aggregates and glass was performed. Their surface free energy calculated on the basis of the sessile drop technique measurements was also determined. The results are shown in Table 3 and Figure 4a–c.

As can be observed, regardless of sulfur/organic copolymer type, its incorporation to bitumen binder significantly improves the adhesive properties to either glass or gravel surface (Table 4). The index of adhesion to the surface of glass increased 3.5 times comparing the unmodified BND 60/90 binder (adhesion 25%) and binder modified with the addition of 2.5% wt of S/DCPD copolymer (adhesion 87%). Generally, adhesion to glass increases with the higher addition of modifier. In turn, adhesion to granite gravel is more prominent with the addition of higher content of sulfur/organic copolymers (2.5% wt) and increase from mark 3 (unmodified bitumen binder and BBMSOC with 1.0% wt) to mark 5 (2.5% wt of copolymers addition). The high improvement of adhesion to both glass and granite gravel surfaces with a small contribution of sulfur/organic copolymers constitutes a very promising technological value for considering it as a potential adhesive promotor in pavement technology.

The presented results of adhesion are also consistent with the results of the surface free energy. Incorporation of sulfur/organic copolymer, regardless of its type, decreases the SFE of modified bitumen binders. It can be observed that the increase of sulfur/organic copolymer content in every example studied results in their lower total SFE, mostly due to the decrease of the polar part of surface energy. Theoretically, the lower the surface free energy of bitumen binders, the higher their adhesion to glass or granite gravel due to better wetting of their surface, as confirmed by the results presented.

#### 3.2.4. FTIR Spectroscopy Analysis of BBMSOC

**Figure 5:**
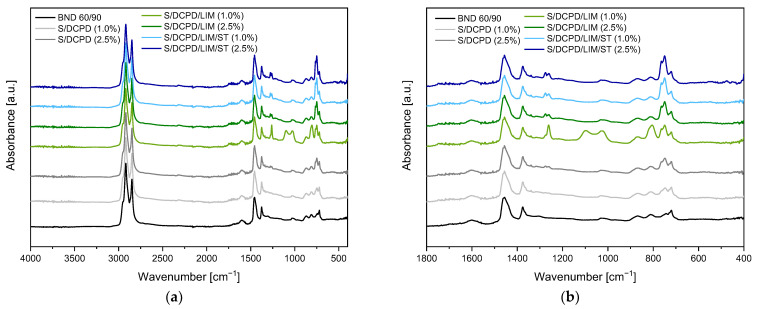
The FTIR spectra of unmodified and sulfur/organic copolymers-modified bitumen binders: (**a**) in the full wavenumber spectrum scope; (**b**) in the wavenumber region of significant changes.

The FTIR spectra of sulfur/organic copolymers-modified bitumen binders as well as unmodified BND 60/90 bitumen in the overall scope (4000–400 cm^−1^) and the range of measurement characteristics for substantial changes are shown in Figure 5a,b.

Although the chemical composition of bitumen materials is very complicated including components with variable molecular weight and structure, the FTIR spectra of bitumen are quite legible. The literature values of wavenumbers corresponding to specific bitumen bands were summarized and tabularized in Table 5.

According to the data shown in Figure 5a,b, the composition of bitumen with sulfur/organic copolymers contains all structural fragments of molecules that are characteristic of petroleum bitumen, namely: vibrations of C–H bonds in the groups CH_3_, –CH_2_–, (CH_2_)_n_, as well as vibrations of C=C bond in the aromatic ring.

However, when a sufficiently large amount of sulfur/organic copolymers is introduced into bitumen, bands at 1275 cm^−1^ and 1255 cm^−1^ can be observed. Moreover, the absorption bands associated with the vibrations of the C–S and S–S groups (which are present when using a sulfur-based modifier) are of low intensity. Thus, it may be concluded that the bands at 1275 and 1255 cm^−1^ correspond to wagging deformational vibrations of RCH_2_S– and R–CH_2_S–S– groups. In the case of pure bitumen, these bands are absent. The bands at 1010–1030 cm^−1^ are vibrations associated with the S=O group, which is present in both the original bitumen (because bitumen contains sulfur) and in the modified products. The band at 755 cm^−1^ corresponds to the trisubstituted aromatic compounds, which indicates the further interaction of bitumen molecules with the modifier.

#### 3.2.5. Thermal Behavior of BBMSOC (TGA-DSC)

**Figure 6:**
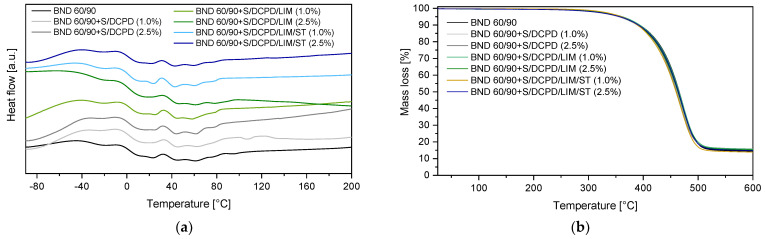
Thermal analysis of bitumen binders modified with sulfur/organic copolymers: (**a**) differential scanning calorimetry (DSC)—second heating scans; (**b**) thermogravimetry (TGA).

The differential scanning calorimetry (DSC) investigation was carried out to examine the general phase structure of bitumen binders as well as to assess their interaction with incorporated sulfur/organic copolymers. In turn, the thermogravimetric (TGA) studies were performed to investigate the thermal stability of unmodified bitumen binder BND 60/90 and to consider the impact of sulfur/organic copolymers addition on their thermal stability. The onset decomposition temperature was established as a temperature required for 5% mass loss during decomposition (T_5%_). Unfortunately, increased interactions between bitumen and the modifiers do not translate into an increase in the thermal stability of the former (T_5%_). The results are shown in Figure 6a,b and the values of temperature thermal effects are summarized in Table 6.

Taking into consideration the chemical structure of bitumen materials, it is well known that bitumen consists of chemically and molecularly diversified components, including high-molecular asphaltenes, exhibiting viscoelastic and viscous properties at elevated temperatures, similar to polymeric materials, and low-molecular (light-weight) fractions including resins, aromatic hydrocarbons and saturates (oils), which are aliphatic hydrocarbons [53].

The measured heat flow curves of the reference sample and bitumen binders modified with sulfur/organic copolymers reveal several thermal effects of bitumen fractions and confirm their complex nature. Typically, at low temperatures (below −20 °C), bitumen materials are in a glassy state, mainly because of the presence of amorphous asphaltenes in their structure [53,54]. In the case of the studied samples, the unmodified bitumen 60/90 possess a glass transition in the temperature interval from c.a. −40 °C with a midpoint of c.a. −30 °C. Analysis of the BBMSOC heat flow curves show similar, however, slightly shifted to lower values Tg.

Generally, incorporation of sulfur/organic copolymers subtly lowered the Tg values of analyzed bitumen, up to 4 °C in the case of the BND 60/90 + S/DCPD/LIM (2.5% wt) sample. This shift toward lower values may be ascribed to the presence in the bitumen of long sulfur/organic copolymer molecules. In bitumen materials, the glass transition is followed by a cold crystallization of low molecular weight saturated hydrocarbons that can order under slow heating [53]. In the samples studied, this transition peak may be spotted in a temperature interval from −20 °C to 10 °C with a midpoint around c.a. 0 °C. For better observation of the above transitions, temperature-modulated DSC (TMDSC) should be applied to analyze the reversing and non-reversing calorimetric signals [53]. Presumably, the cold crystallization of low-molecular weight fraction starts earlier and overlaps with the glass transition of high-molecular weight hydrocarbons. Further heating leads to the mild melting of the crystals and afterward recrystallization of high-molecular weight hydrocarbons is observed, due to increased mobility of the molecules, in the temperature range from c.a. 23 °C to 42 °C. The enthalpy of the latter varies from 0.95 J/g to 1.46 J/g, depending on the sample. The melting of this ordered phase can be found around 60 °C. Above this temperature, no more calorimetric signals are observed in conventional DSC curves such as a high-temperature glass transition of resin-asphaltene amorphous phase [53]. What is more, the endothermic signals in the temperature region c.a. 90–110 °C correspond to the melting of sulfur/organic copolymers that are not spotted on the DSC curves of BBMSOC. This may suggest that chemical interactions between elemental sulfur and sulfur/organic macromolecules with bitumen components might be formed.

The TGA analysis shows that the addition of sulfur/organic copolymers does not affect the thermal stability of the bitumen. The analyzed samples exhibit similar onset temperatures of thermal decomposition c.a. 360 °C. The thermal decomposition kinetics is smooth and very similar for all the samples studied.

## 4. Conclusions

The presented study showed that the sulfur/organic copolymers obtained by the facile method so-called “inverse vulcanization” can be potentially applied as modifiers for bitumen binders. Investigation of the modification impact onto the overall properties of bitumen binders shows that the bitumen binders modified with sulfur/organic copolymers are in general less sensitive to higher temperatures (higher softening point) and also more resistant to permanent deformations (lower penetration depth) without intrusive deterioration of parameters at lower temperatures. A consistent trend of softening point and penetration changing of bitumen binders has been presented by Elkholy et al. [30] with the use of elemental sulfur-olefin hydrocarbons/bituminous residue-derived polysulfides. What is more, the lower increase of softening point after short-term RTFOT aging in comparison to raw bitumen indicates a higher resistance to aging processes of the modified bitumen systems. Nanoindentation measurements revealed that the modified bitumen binders, particularly modified with S/DCPD/LIM and S/DCPD/LIM/ST copolymers, exhibit more elastic behavior (higher participation of elastic deformation), which, in combination with their lower penetration, constitutes promising materials from the point of view of their application as attractive rutting preventing additives. Modification of the bitumen binder BND 60/90 with the use of a small contribution of sulfur/organic copolymers results in significantly higher adhesive properties to both glass and granite gravel surfaces. In the best example, the adhesion to glass increased from 25% (raw BND 60/90) up to 87% (for 2.5 wt % addition of S/DCPD copolymer). Comparing all the studied samples, the most promising modification system seems to be S/DCPD (2.5% wt), which falls within all required parameters of standardized BMPA 60/90-53 and provides ultimate adhesion both to glass and gravel. Limitations of the tested modifiers ought to be taken into consideration, because some of the modifiers did not fall with all the standardized demands. The surprising lack of research works concerning the utilization of inverse vulcanization or other high-molecular sulfur-containing products in the field of bitumen/paving industry creates a research gap that should be fulfilled in the future by researchers bringing the progress in the both synthetic and application field of the presented work. This study proved that the improvement of the bitumen binders’ performance can be achieved by the addition of sulfur/organic copolymers. This opens the possibility to raise the level of standards applied in the bitumen/paving industry.

## Figures and Tables

**Table 1:** Copolymerization conditions and composition of sulfur/organic copolymers as well as their organoleptic features.

Sample Symbol	Sulfur/Organic Comonomer(s) Ratio (wt %)	Reaction Temperature (°C)	Reaction Time ^1^ (h)	Organoleptic Viscosity ^2^
S/DCPD	90.9/9.1	140	3	Low
S/DCPD/LIM	87.0/8.7/4.3	140	3	Moderate
S/DCPD/LIM/ST	83.3/8.3/4.2/4.2	140	3	Moderate to High

^1^ Reaction time measured after the total dripping of organic comonomer(s) into liquid sulfur at 140 °C; ^2^ Determined while pouring away the copolymerization mixture to molds; Abbreviations: DCPD—dicyclopentadiene, LIM—limonene, ST—styrene.

**Table 2:** Thermogravimetric and DSC characteristic parameters of the sulfur/organic copolymers. Elemental sulfur results were added for comparison.

Sample Symbol	T_PT_ ^1^ [°C]	ΔH_PT_ ^2^ [J/g]	T_5%_ ^3^ [°C]
Elemental sulfur (S_8_)	108 ^a^; 120 ^b^, 170 ^c^	14.5 ^a^; 44.9 ^b^; 7.6 ^c^	320
S/DCPD	108 ^d^	27.9 ^d^	245
S/DCPD/LIM	104 ^d^	21.2 ^d^	244
S/DCPD/LIM/ST	98 ^d^	14.7 ^d^	245

^1^ Temperature of characteristic phase transitions; ^2^ Enthalpy of characteristic phase transitions; ^3^ Decomposition temperature at 5% mass change; ^a^ Transition of orthorhombic sulfur to monoclinic sulfur; ^b^ Sulfur crystals melting; ^c^ Ring-opening polymerization of sulfur molecules; ^d^ Fused melting.

**Table 3:** Performance characteristic of the BBMSOC.

Property	BND 60/90	BMPA 60/90-53	S/DCPD		S/DCPD/LIM		S/DCPD/LIM/ST	
			1% wt	2.5% wt	1% wt	2.5% wt	1% wt	2.5% wt
Homogeneity (-)	n.s. ^1^	^2^	+	+	+	+	+	+
Softening Point (°C)	46	≥53	49	53	49	53	49	51
Ductility at 25 °C (cm)	63	≥15	61	42	60	35	58	40
Penetration at 25 °C (0.1 mm)	70	61–90	68	62	62	50	64	46
Penetration index (-)	−1.5	n.s. ^1^	−0.76	0.03	−0.96	−0.31	−1.5	−1.15
Fraas breaking point (°C)	−18	≤−20	−18	−19	−18	−18	−18	−19
Adhesion to gravel (mark)	3	≥3	3	5	3	4	3	5
Adhesion to glass (%)	25	≥20	43	87	50	65	54	80

^1^ not standardized; ^2^ clots and polymer particles should not be observed.

**Table 4:** Changes in performance characteristics of bitumen after RTFOT.

Property	BND 60/90		S/DCPD (2.5% wt)		S/DCPD/LIM (2.5% wt)		S/DCPD/LIM/ST (2.5% wt)	
	Unaged	RTFOT	Unaged	RTFOT	Unaged	RTFOT	Unaged	RTFOT
Softening Point (°C)	46	52	53	56	53	56	51	55
Change in Softening Point (°C)	-	6	-	3	-	3	-	4
Penetration at 25 °C (0.1 mm)	70	55	62	48	50	30	46	24
Residual Penetration (%)	-	79	-	77	-	60	-	52
Change in Weight (wt %)	-	0.030	-	0.055	-	0.190	-	0.237

**Table 5:** Values of wavenumber attributed to the characteristic groups present in bitumen materials [49,50,51,52].

Wavenumber [cm^−1^]	Characteristic of the Peak
2950	Asymmetric stretching vibrations ν (C–H) in CH_3_– group
2920	Asymmetric stretching vibrations ν (C–H) in CH_2_– group
2850	Symmetric stretching vibrations ν (C–H) in CH_2_– group
1600	Stretching vibrations ν (C=C) in aromatic rings
1455	Asymmetric bending vibrations δ (C–H) in CH_2_– group
1375	Symmetric bending vibrations δ (C–H) in CH_3_– group
1275	Wagging deformational vibrations ω (C–S and S–S) in RCH_2_S- and RCH_2_S-S-group
1255	
1010–1030	Rocking vibrations (bending with torsion) ν (S=O) in sulfoxide group
870	Deformational vibrations of CH– group in 1,2,4-trisubstituted derivatives of benzene
810	Deformational vibrations of CH– group in tetrasubstituted derivatives of benzene
755	Deformational vibrations of CH– group in trisubstituted derivatives of benzene
745	Deformational vibrations of CH– group in 1,2,3-trisubstituted derivatives of benzene
720	Rocking vibrations of CH_2_– group in 1,3-disubstituted derivatives of benzene

**Table 6:** Thermogravimetric and DSC characteristic parameters of sulfur/organic copolymers-modified bitumen binders.

Sample Symbol	T_g_ ^1^ [°C]	T_g_ ^2^	T_5%_ ^3^ [°C]
BND 60/90	−39.6	−30.2	360
BND 60/90 + S/DCPD (1.0%)	−34.9	−31.7	353
BND 60/90 + S/DCPD (2.5%)	−32.4	−29.8	360
BND 60/90 + S/DCPD/LIM (1.0%)	−39.2	−32.0	362
BND 60/90 + S/DCPD/LIM (2.5%)	−43.9	−34.0	357
BND 60/90 + S/DCPD/LIM/ST (1.0%)	−41.7	−31.8	360
BND 60/90 + S/DCPD/LIM/ST (2.5%)	−39.8	−32.7	357

^1^ Onset temperature of glass transition; ^2^ Midpoint temperature of glass transition; ^3^ Decomposition temperature at 5% mass change.

## Data Availability

The data presented in this manuscript are available on request from the corresponding authors.
